# Ferritin heavy chain protects the developing wing from reactive oxygen species and ferroptosis

**DOI:** 10.1371/journal.pgen.1008396

**Published:** 2019-09-30

**Authors:** Simone Mumbauer, Justine Pascual, Irina Kolotuev, Fisun Hamaratoglu

**Affiliations:** 1 Center for Integrative Genomics, University of Lausanne, Lausanne, Switzerland; 2 Electron Microscopy Facility, University of Lausanne, Lausanne, Switzerland; 3 School of Biosciences, Cardiff University, Cardiff, United Kingdom; German Cancer Research Center (DKFZ), GERMANY

## Abstract

The interplay between signalling pathways and metabolism is crucial for tissue growth. Yet, it remains poorly understood. Here, we studied the consequences of modulating iron metabolism on the growth of *Drosophila* imaginal discs. We find that reducing the levels of the ferritin heavy chain in the larval wing discs leads to drastic growth defects, whereas light chain depletion causes only minor defects. Mutant cell clones for the heavy chain lack the ability to compete against *Minute* mutant cells. Reactive oxygen species (ROS) accumulate in wing discs with reduced heavy chain levels, causing severe mitochondrial defects and ferroptosis. Preventing ROS accumulation alleviates some of the growth defects. We propose that the increased expression of ferritin in *hippo* mutant cells may protect against ROS accumulation.

## Author summary

Despite being vital, the role of metals in biology is often overlooked. Specifically, how iron storage contributes to development remains unclear. Here, we dissected the function of the cellular iron storage complex, Ferritin, during development of a model organ, the fly wing. We took a genetic approach to uncover the role of both the heavy and light chains of Ferritin. Targeting the heavy chain consistently produced drastic growth defects in larval discs and adult wings. Moreover, lower levels of the heavy chain led to formation of reactive oxygen species (ROS) and preventing ROS accumulation alleviated the accompanying growth defects. We also observed hallmarks of ferroptosis, an iron dependent non-apoptotic cell death, upon knockdown of the heavy chain. By contrast, reducing the levels of the light chain was tolerable. This is surprising because the individual components of Ferritin were thought to function exclusively as part of the complex. Yet, the heavy chain alone encompasses the ferroxidase center, which is essential for iron loading. All together, we propose that the Ferritin heavy chain functions as an antioxidant and protects the developing organs from ferroptosis.

## Introduction

Iron is indispensable for the well-being of cells, from prokaryotes to eukaryotes. Many key enzymes involved in ATP production, photosynthesis, and DNA biosynthesis require iron-sulphur clusters to function [1, 2]. An excess of iron can also be deleterious for cells, thus it is critical to maintain iron homeostasis [1]. Ferrous iron (Fe^2+^) is readily converted to the ferric iron (Fe^3+^) under aerobic conditions or *via* the Fenton reaction. The latter reaction generates reactive oxygen species, which damage biological macromolecules. Iron-binding proteins, such as ferritin, ensure that iron levels in a cell are tightly controlled. The insect ferritin complex is formed by 24 subunits of the heavy and the light chain proteins in a 1:1 ratio; it is the main iron storage facility of the cell [3]. Two adjacent genes named *Ferritin 1 Heavy Chain Homolog (Fer1HCH)* and *Ferritin 2 Light Chain Homolog (Fer2LCH)* encode the components of this complex in *Drosophila*. Being next to each other in the genome, these two genes share regulatory elements for coordinated synthesis [4]. The function of ferritin has been studied in different contexts and the emerging view is that its main role is to sequester iron and keep it in a non-toxic form [5]. This potentially protects the cells from apoptosis and gives them a proliferative advantage. For example, an oceanic diatom, *Pseudo-nitzschia granii*, that expresses ferritin can undergo several more cell divisions in the absence of iron than another comparably sized species that lacks a functional ferritin gene [6]. In hard ticks, ferritins are essential for protection from iron-mediated oxidative stress during blood feeding [7]. Similarly, induction of ferritin expression during heart ischaemia/reperfusion in rats is protective against oxidative damage [8]. Moreover, iron-loaded ferritin is an essential mitogen in the medium for cultured *Drosophila* cells [9]. Here, we took a genetic approach and dissected the involvement of the ferritin subunits in growth control in *Drosophila*.

A major player in growth control in *Drosophila* is the Hippo pathway, a modulator of cell proliferation and apoptosis with close ties to human cancer. Ten percent of all sequenced human cancers have mutations in Hippo components [10]. Originally discovered in *Drosophila*, the pathway and its functions are conserved [11–14]. The pathway is named after the Hippo kinase, which once inactivated leads to excessive growth of epithelial tissues mainly by increasing the number of undifferentiated cells and making them resistant to apoptotic stimuli. The Hippo kinase is regulated by various upstream inputs and relays information from the extracellular milieu into transcriptional decisions. Hippo activates the Warts kinase, which in turn inhibits the transcriptional co-activator Yorkie (Yki, YAP/TAZ in mammals) [15]. In the absence of Hippo and thus Warts activity, Yki is free to translocate into the nucleus. Together with the transcription factor Scallopped (Sd) and others, Yki induces expression of genes driving proliferation (cyclin E, string), cell growth (myc, bantam), and apoptotic resistance (DIAP1) [11]. Here, we describe a novel outcome of active Yki that links Hippo signalling to iron metabolism. We found that in *warts* mutant discs, the synthesis of both subunits of the ferritin complex is stimulated. We show that the enhanced levels of especially *Fer1HCH* contribute to the excess growth induced in the absence of Hippo activity. This might be due to its protective effect against ROS formation and ferroptosis.

## Results

### Ferritin is deregulated in *warts* mutant discs and contributes to excess growth

**Fig 1:**
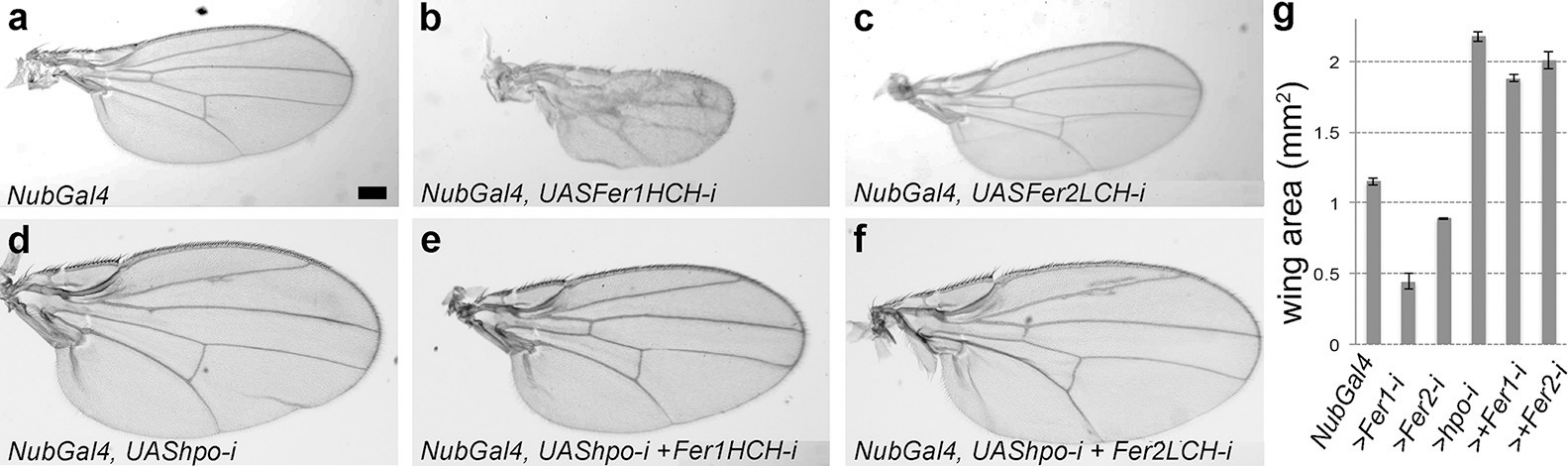
Knockdown of ferritin subunits causes growth defects and partially supresses overgrowth induced upon *hippo* knockdown. **(a-f)** Representative male wings of indicated genotypes. (b) *NubGal4 > VDRC 49536*, (c) *NubGal4 > VDRC 14491* **(g)** Quantification of wing areas. Minimum 8 wings were measured for each genotype and the average wing areas were plotted with standard error. The differences are significant between all pairs (student’s t-test, p < 0.05). Also see the related S2 Fig. All images are shown at the same scale and the scale bar in (a) corresponds to 100 microns.

We have recently profiled the transcriptional landscape in the absence of the Warts kinase in the larval wing tissue [16, 17]. To determine which transcriptional changes contribute to the growth phenotypes, we carried out an RNAi screen with the top 120 upregulated genes, with the cut off of minimum 2,3-fold increase, in *warts* mutant discs. As multiple *Fer1HCH* RNAi lines led to growth defects and modified Hippo phenotypes, we decided that the potential role of *Fer1HCH* in growth control was worthy of further study. Notably, *Fer1HCH* was not among the very highly induced target genes (S1A Fig). Nevertheless, qRT-PCR analysis confirmed the modest upregulation for both the heavy and the light subunits (S1B and S1C Fig). We wondered whether this apparent modification of iron metabolism machinery contributed to the overgrowth observed in wings with low Hippo activity. First, we tested multiple UAS-RNAi lines to modulate the levels of the heavy and the light chains of ferritin using the wing-specific Nub-Gal4 driver (Figs 1 and S2A). Knockdown of the heavy chain led to dramatic phenotypes ranging from small wings to no wings to lethality, depending on the RNAi line used (Figs 1A, 1B and S2A). Knockdown of the light chain, on the other hand, led to very mild growth defects (Figs 1C and S2A). Next, we crossed all UAS-RNAi lines to a *hippo* knocked down background. Knockdown of either subunit led to a modest but consistent suppression of the overgrowth induced upon reduction of Hippo activity (Figs 1D–1F, 1G and S2B).

**Fig 2:**
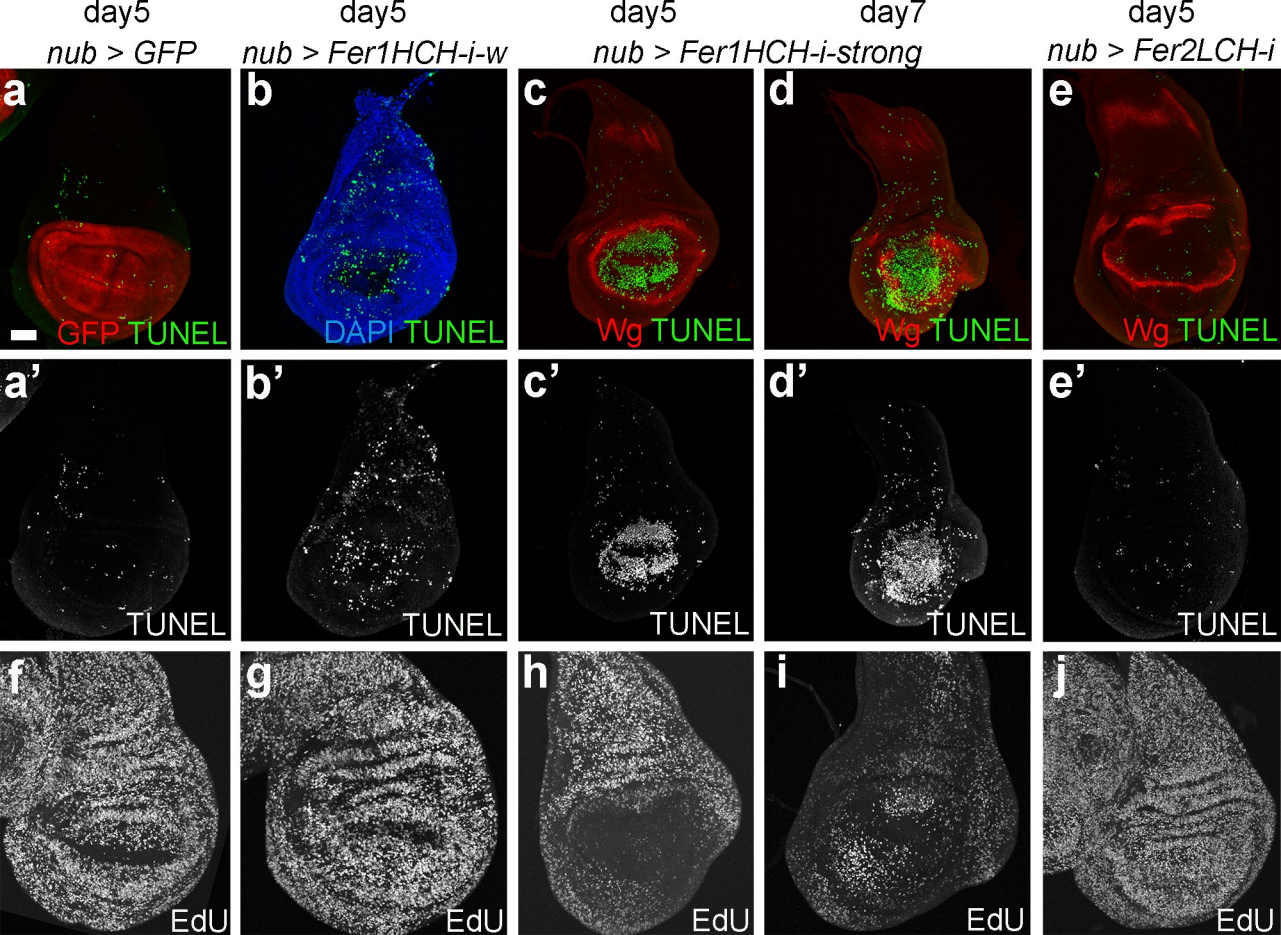
Knockdown of the ferritin heavy chain in the pouch influences proliferation and induces excessive cell death. Representative third instar wing imaginal discs with *Nub-Gal4* driven overexpression of GFP (a and f), the weak *Fer1HCH–RNAi* (49536 (b and g)), the strong *Fer1HCH -RNAi* (12925 (c-d, h-i)) and *Fer2LCH -RNAi* (14491 (e and j)), in the pouch, at indicated time points. (a’-e’) shows the TUNEL staining in gray, and (f-j) shows EdU staining in gray. Wingless (Wg) staining (red in c-e) surrounds the pouch and marks the D/V boundary. All images are shown at the same scale and the scale bar in (a) corresponds to 50 microns.

We reasoned that since the ferritin complex is formed from Fer1HCH and Fer2LCH in equal parts, removing either component should result in the disruption of the iron-storage complex. Surprisingly, depleting *Fer1HCH* transcripts with different wing Gal4 drivers consistently resulted in smaller wings or lethality whereas targeting *Fer2LCH* had very mild consequences (Figs 1, 2, S2 and S3). These observations suggested that the heavy chain may have a function independent of the complex or that knockdown of the light chain is not sufficient to disrupt the complex. As lowering *Fer1HCH* levels led to dramatic growth defects in adult wings, next we examined the larval discs. When we used the stronger RNAi lines (VDRC 102406 and 12925) to knockdown the heavy chain with the posterior *hh-Gal4* and anterior *ci-Gal4* drivers, majority of the animals died before the third instar stage. We found few survivors, and these had tiny discs (S3C and S3G Fig). The use of weaker RNAi lines (VDRC 49536 and 49567) and the *hh-Gal4* driver to knockdown the heavy chain resulted in smaller discs and even smaller regions where the RNAi construct was expressed (S3B and S3F Fig). Hence, knockdown of the heavy chain has a negative effect on growth both autonomously and cell non-autonomously. Knockdown of the light chain produced, if any, very weak effects in larval discs (S3D and S3H Fig).

With the pouch-specific *Nub-Gal4* driver (Fig 2A, expression pattern in red), which comes on later than *hh* and *ci-Gal4* drivers, it was possible to obtain viable third instar larvae when the strong *UAS-Fer1HCH-RNAi* lines were used. We used this setup to assess the cell proliferation and cell death patterns in larval discs upon ferritin knockdown. Knockdown of the heavy chain in the pouch with the weak RNAi line led to an increase in the number of dying cells (Fig 2A and 2B), but did not influence the EdU pattern (Fig 2F and 2G). Fer1HCH knockdown with the stronger RNAi lines extended the larval period from 5 to 8 days. At larval day 5, these discs had nearly normal patterning, judged by Wg staining (Fig 2C). By day 7, the pattern in the discs was heavily disrupted and the pouch was smaller (Fig 2D). Very few cells undergo apoptosis under normal conditions during disc development (Fig 2A’). There was, however, a massive amount of cell death in the pouch when the ferritin heavy chain was knocked down with the stronger RNAi lines (Fig 2C–2D’). Additionally, we found lower levels of EdU incorporation suggesting lower proliferation rates in the pouch (Fig 2H and 2I, compared to uniform EdU pattern of a normal disc in Fig 2F). Notably, the surviving adults of this genotype (nub > 12925) have no wings. We conclude that the disc initially forms and then the cells in the pouch eventually die leading to the absence of wings in the surviving adults. In stark contrast, knockdown of the light chain displayed no discernable effects on cell proliferation and cell death patterns (Fig 2E and 2J).

In addition, we analysed the cell proliferation and cell death patterns upon heavy and light chain knockdown using the early, posterior *hh-Gal4* driver (S3A–S3H’ Fig). As expected, EdU incorporation was uniform in control discs (S3A Fig). When *Fer1HCH* was reduced in the posterior compartment using the weak RNAi line, we observed no cell-autonomous effects on EdU incorporation (S3B Fig). Only a few, tiny discs were obtained when the strong RNAi lines against the heavy chain were used and the stainings of such discs were not informative (S3C and S3G Fig). Next, we examined the pattern of cell death. Reducing ferritin heavy chain levels in the posterior compartment with the weak RNAi line led to cell autonomous induction of the activated caspase Dcp-1 (S3E and S3F Fig), indicating higher numbers of apoptotic cells consistent with the TUNEL staining shown in Fig 2B. Hh-driven knockdown of the light chain did not disrupt the proliferation or the cell death patterns in the wing disc (S3D and S3H Fig). Overall, Fer2LCH knockdown does not produce evident phenotypes, unlike the Fer1HCH knockdown.

### *Fer1HCH* mutant cells are super-losers

Furthering our results with the RNAi lines, we studied the function of the ferritin subunits in the larval discs using established, reportedly null, loss-of-function alleles [18–20]. We have recombined *Fer1HCH*^*451*^ *and Fer2LCH*^*35*^ onto FRT chromosomes to perform mosaic analysis since both alleles are lethal at the L1 larval stage [18]. We also used a small deletion that removes the 5’ regions of both genes as a double mutant null allele, referred to as *Df(3R)Fer* (a.k.a. *Fer*^*x1*^*)* [20]. Surprisingly, heat-shock induced (at day 2 or 3) patches that are mutant for either gene or doubly mutant, were by eye comparable in size to their wild-type twin spots and the overall disc size was not affected. This indicated that either the RNAi lines displayed a phenotype due to an off-target effect, or that the transcripts are very stable and are not degraded quickly enough. To test the latter possibility, we induced clones earlier during development and provided a longer period for depletion.

**Fig 3:**
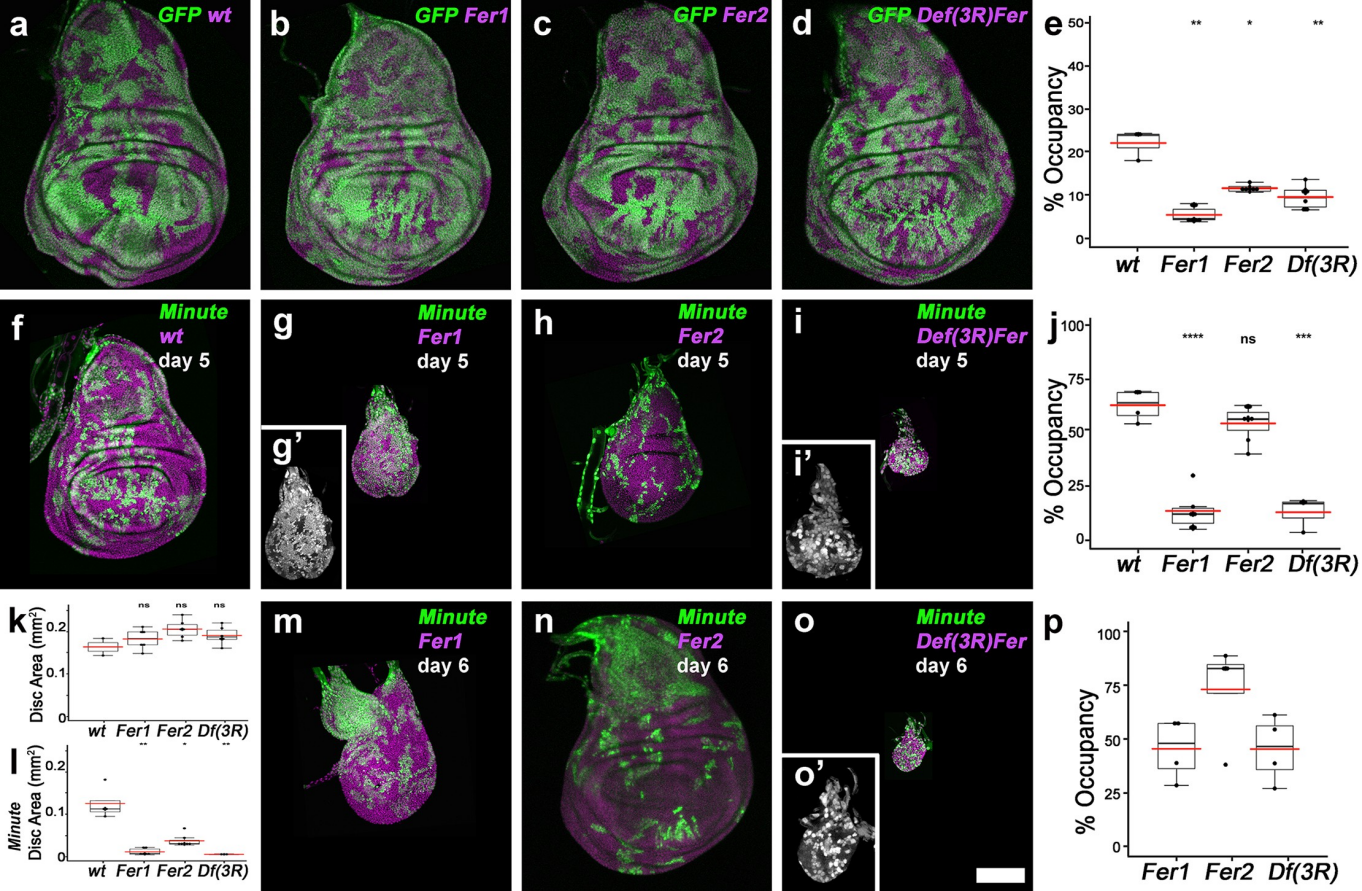
Cells mutant for the ferritin heavy chain cannot compete against *Minute* cells. Mosaic analysis of *Fer1HCH*^*451*^, *Fer2LCH*^*35*^ and *Df(3R)Fer* mutant chromosomes in wild-type (a-e, k) and *Minute* backgrounds (f-j and l-p). The disc size quantifications shown are from day 5 mosaic discs in a wild-type (k) and Minute backgrounds (l). Statistical significance is indicated as ns: p>0.05, *: p≤0.05, **: p≤0.01, ***: p≤0.001, ****: p≤ 0.0001. (g’) shows the GFP channel alone in gray. All discs are shown at the same scale except for the insets (i’) and (o’); they are magnified (2x zoom) and show only the GFP channel in gray. Scale bar in (o) is 100 microns. Genotypes are: a) *y ubx-flp; FRT82B ubiGFP / FRT82B* b) *y ubx-flp; FRT82B ubiGFP / FRT82B Fer1HCH*^*451*^ c) *y ubx-flp; FRT82B ubiGFP / FRT82B Fer2LCH*^*35*^ d) *y ubx-flp; FRT82B ubiGFP / FRT82B Df(3R)Fer* f) *y ubx-flp; FRT82B M(3) ubiGFP / FRT82B* g-m) *y ubx-flp; FRT82B M(3) ubiGFP / FRT82B Fer1HCH*^*451*^ h-n) *y ubx-flp; FRT82B M(3) ubiGFP / FRT82B Fer2LCH*^*35*^ i-o) *y ubx-flp; FRT82B M(3) ubiGFP / FRT82B Df(3R)Fer*.

To induce clones earlier in development, we used the *ubx-FLP* and *ey-FLP* drivers that are active in the wing and eye discs, respectively (Figs 3 and S4). We quantified the overall tissue size and the GFP negative area occupied by the mutant cells. Supporting the transcript stability hypothesis, inducing clones earlier in development revealed proliferation defects for *Fer1HCH*^*451*^ and *Df(3R)Fer* mutant cells (Figs 3A–3D and S4A–S4D). In both the wing and eye discs, cells lacking the ferritin heavy chain occupied significantly smaller areas compared to the wild-type cells (Figs 3E and S4I). *Fer2LCH*^*35*^ mutant cells occupied larger areas than the cells lacking the heavy chain, but were not as proliferative as the wild-type cells in the wing (Fig 3C and 3E). Eye discs mosaic for *Fer2LCH*^*35*^ were nearly identical to discs with wild-type clones in all aspects (S4A, S4C and S4I Fig). Even when the slow-growing mutant cells occupied part of the disc, the overall disc size was maintained, presumably due to compensatory proliferation of the wild-type cells in the tissue (Fig 3K).

Next, we wanted to give our mutant cells a growth advantage and allow them to occupy larger areas in the tissue using the *Minute* technique [21]. In the presence of *Minute* cells (GFP-positive), wild-type cells occupied the majority of the disc and *Fer2LCH* mutant cells behaved very similarly (Figs 3F, 3H, 3J, S4E, S4G and S4J). Wing discs that are mosaic for *Minute* and *Fer2LCH* clones were smaller than control discs on day 5 (Fig 3H). Nevertheless, these animals stayed as larvae for an additional day, before the discs reached a normal size and gave rise to progeny with normal wings (Fig 3N). Therefore, even when *Fer2LCH* mutant cells occupied the majority of the disc, its development was not compromised, suggesting that Fer2LCH function is largely dispensable for disc development. This was, however, not the case for Fer1HCH. Surprisingly, *Fer1HCH* and *Df(3R)Fer* mutant cells failed to outcompete *Minute* cells (Figs 3J and S4J). Mutant cells were able to occupy at best nearly half the disc at day 6 (Fig 3P). These discs were very small at all ages (Figs 3L, 3M, 3O, S4F and S4H). The presence of *Fer1HCH*, *Minute* mosaic discs extended the larval phase and the mutant cells occupied larger areas at day 6 and 7, but the discs stayed small and only a small proportion of the larvae were able to pupate and formed tiny pupae. Hence, wild-type cells–in contrast to *Minute* cells–can compensate for low ferritin levels in mosaics and maintain the overall tissue size and pattern.

Next, we evaluated apoptosis in this set-up using TUNEL staining. Discs containing *Fer1HCH* or *Fer1*, *Fer2* double mutant *Df(3R)* cell clones showed highly elevated levels of apoptotic staining. The TUNEL signal was not specifically associated with one population. Instead, both heavy chain mutant and *Minute* cells showed increased apoptotic signal and the resulting discs were small. In contrast, discs with *Fer2LCH* mutant clones had only a few TUNEL positive cells similar to discs with wild-type clones (S3I Fig). This result further indicated that low Fer1HCH levels are unfavourable for the cell, whereas the tissue can tolerate low Fer2LCH levels.

### Ferroptosis contributes to the *Fer1HCH* mutant phenotype

Upon knockdown of ferritin heavy chain with various drivers, we observed excessive cell death and caspase activation (Figs 2B–2D, S3E and S3F). Similarly, discs mosaic for *Fer1HCH* and *Minute* cells displayed increased levels of cell death (S3I Fig). These results prompted us to ask whether blocking apoptosis could rescue some of the growth defects. To this end, we co-expressed various inhibitors of apoptosis: p35, DIAP1 and RHG-miRNA, together with *Fer1HCH* RNAi lines using *nub-Gal4* and *hh-Gal4* drivers. Preventing apoptosis with these treatments did not visibly supress the reduced wing and disc size phenotypes observed upon knockdown of *Fer1HCH*. Next, we co-expressed inhibitors of apoptosis in clones together with the strong *Fer1HCH* RNAi line, such clones were readily eliminated from the tissue and the surrounding cells compensated for their absence (S5A and S5B Fig). Co-expression of p35 or DIAP1 gave a mild rescue (significant with p≤0.05) (S5C, S5D and S5F Fig). In contrast, Yki co-expression was effective at rescuing the clone viability and size (S5E and S5F Fig). However, keeping the clones in the tissue adversely affected the tissue well-being; such discs were consistently small (S5F Fig). Overall, the rescue obtained with inhibiting apoptosis was context dependent and at best incomplete. These observations suggested that cells might be dying also via caspase-independent cell death pathways. One such pathway associated with iron metabolism is ferroptosis, an iron dependent, non-apoptotic, regulated cell-death [22]. The term ferroptosis was coined relatively recently upon the discovery that iron chelators could block the non-apoptotic cell death induced upon treatment with a small Ras inhibitor, erastin [23]. Cells deprived of amino acids, specifically cysteine, which is limiting for glutathione biosynthesis, also undergo ferroptosis [24, 25]. Ferroptosis has unique morphological characteristics; in electron micrographs, mitochondria are shrunken and damaged, whereas the nuclei remain intact.

**Fig 4:**
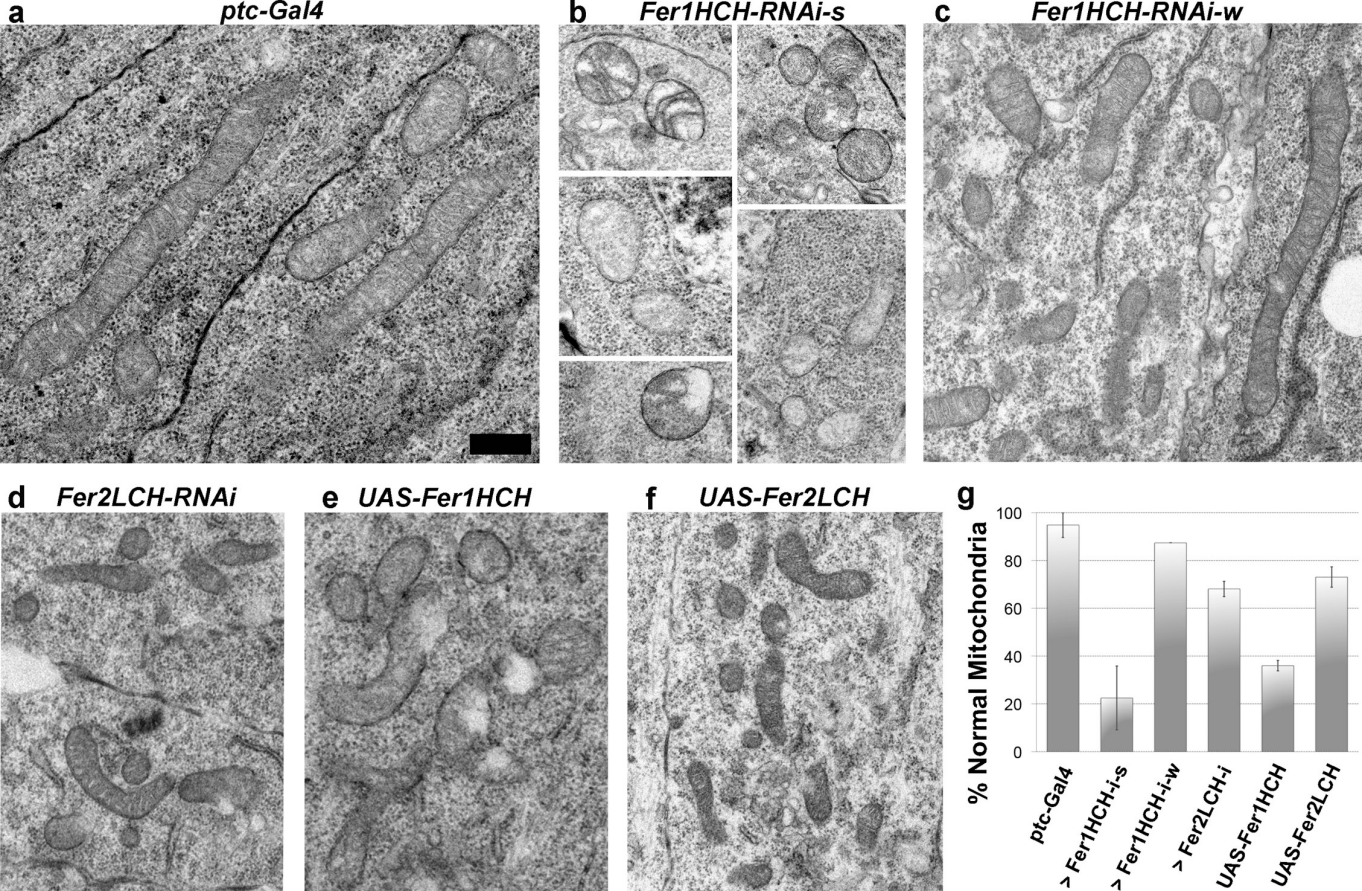
Ferritin knockdown leads to drastic mitochondrial defects, reminiscent of ferroptosis. Representative TEM images of mitochondria from **(a)** control discs and discs with *ptc-gal4* driven **(b)** *Fer1HCH* knockdown with a strong RNAi line (102406), **(c)** the weaker RNAi-line (49536), **(d)** *Fer2LCH* knockdown (14491), **(e)** Fer1HCH overexpression and **(f)** Fer2LCH overexpression. **(g)** Quantification of mitochondria based on morphology. Intact mitochondria with their cristae visible were considered normal. Also see S6E–S6F Fig for examples for normal and abnormal mitochondria. We scored all mitochondria in at least two composites from different discs (min 74, max 2288 mitochondria per genotype). Error bars represent standard error. All images are shown at the same magnification. Scale bar in (a) is 500nm.

We examined mitochondrial morphology using Transmission Electron Microscopy (TEM) upon modifications to ferritin levels in wing discs. In control discs, the mitochondrial double membrane and cristae were clearly visible. These organelles were well defined, undamaged and could be seen as long or circular in the cross sections depending on their position within the tissue (Fig 4A). Upon *Fer1HCH* knockdown using a strong RNAi line, we found severe defects in a vast majority of the mitochondria in the tissue (Fig 4B and 4G). Out of 250 mitochondria scored, only 12 were long and another 30 looked almost normal, the rest were damaged (nearly 80%, Fig 4G). We used the *ptc-Gal4* driver with the expectation that we may see a gradient of phenotypes, most severe in the central stripe where *ptc* is expressed and no phenotypes at the periphery. However, the defects were severe and throughout the tissue. Thus, the effect was clearly non-cell autonomous (S6B and S6D Fig). Knockdown of Fer1HCH using the weaker RNAi line did not cause drastic changes in mitochondrial morphology (Fig 4C). Interestingly, excess Fer1HCH also led to mitochondrial defects; the frequency and the severity of the defects were milder compared to *Fer1HCH* knockdown and the overall disc morphology was normal (Figs 4E, 4G, S6C and S6D). Instead, the mitochondria in discs with Fer2 overexpression or knockdown had significantly fewer defects, the most common amongst them being indiscernible cristae (Figs 4D, 4F, 4G, S6A and S6D). Hence, the mitochondrial defects seen in discs with strong *Fer1HCH* knockdown and to a lesser extent with Fer1HCH ectopic expression are reminiscent of ferroptosis.

Another hallmark of ferroptosis is the accumulation of ROS in the tissue. ROS are readily generated as products of various metabolic activities, including the Fenton reaction. Excess ROS leads to lipid peroxidation, which in turn leads to ferroptotic death via a poorly defined process [25, 26]. Thus, we tested whether ROS accumulation contributes to the growth defects of discs with reduced ferritin levels. Transcriptional induction of a group of antioxidant enzymes, among which are the Glutathione S-transferases (Gst), occur in response to oxidative stress and can report ROS formation. A *GstD1-GFP* transgene is commonly used in *Drosophila* as a ROS reporter [27].

**Fig 5:**
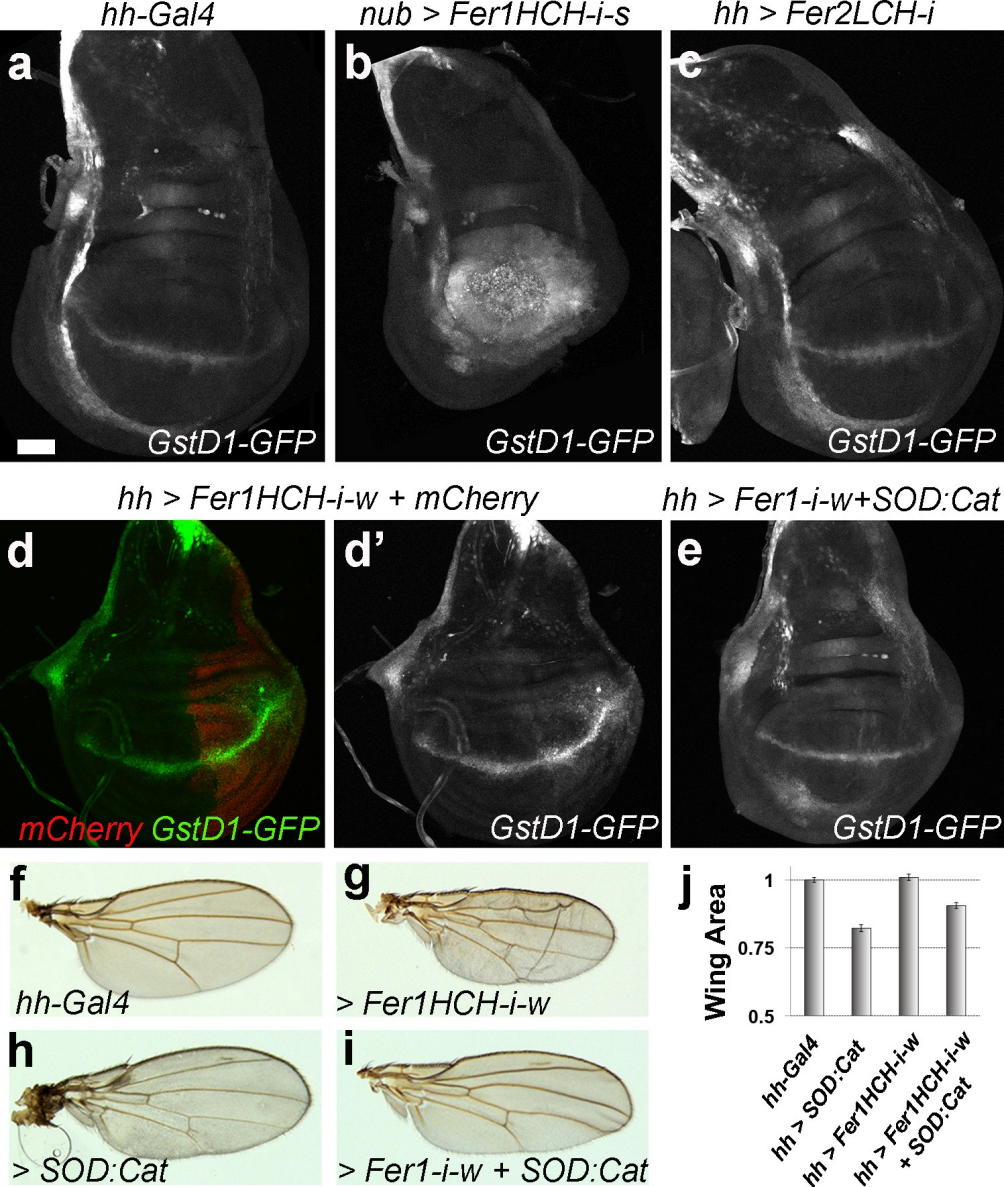
Low levels of the Ferritin heavy chain cause ROS accumulation. **(a-e)** Representative wing discs of indicated genotypes. Dorsal is up, anterior is to the left. *hh-Gal* is expressed in the posterior compartment and *nub-Gal4* is expressed in the pouch (used in panel b). (d’) shows the *GstD1-GFP* signal alone of the disc shown in panel (d). All discs are from day 5 larvae with the exception of panel (b), which shows a day 8 disc. **(f-i)** Representative wings of indicated genotypes and **(j)** quantification of their areas normalized to control wings (minimum 10 wings per genotype) are shown. Error bars represent standard error. P-values for t-tests are: *hh-Gal4* vs *Fer1HCH-i-w* (p≤ 0.0001); *hh-Gal4* vs *Fer1HCH-i-w* + *SOD*:*Cat* (p≤ 0.01); *Fer1HCH-i-w* vs *Fer1HCH-i-w* + *SOD*:*Cat* (p≤ 0.001). All discs and all wings are shown at the same scale. Scale bars in (a) and (f) are 50 and 100 microns respectively.

*GstD1-GFP* accumulated in the pouch region of discs where we knocked down *Fer1HCH* with the stronger RNAi line (12925) (Fig 5A and 5B). Similarly, knockdown of *Fer1HCH* with the weaker RNAi line (49536) led to excess, albeit weaker, ROS formation (Fig 5D and 5D’). In contrast, *Fer2LCH* knockdown did not cause ROS accumulation in the tissue (Fig 5C). Next, we asked whether ROS formation contributed to the growth defects upon *Fer1HCH* knockdown. We prevented ROS accumulation in cells with reduced *Fer1HCH* by co-overexpression of two enzymes that degrade reactive oxygen species, Superoxide Dismutase (SOD) and Catalase (Cat) using the UAS-SOD:Cat transgene [28, 29]. This treatment indeed reversed the *GstD1-GFP* accumulation in discs with reduced *Fer1HCH* levels (Fig 5E). Knocking down *Fer1HCH* with hh-gal4 led to formation of smaller adult wings (Fig 5G and 5J). On its own, *SOD*:*Cat* expression didn’t influence the overall size, but did cause mild darkening of the wings (Fig 5H and 5J). SOD:Cat overexpression rescued the small wing size phenotype caused by *Fer1HCH* depletion to near wild-type size (Fig 5I and 5J, p≤ 0.001). These findings demonstrate the significance of ROS formation in the events leading to a decrease in size in *Fer1HCH* knockdown tissues.

**Fig 6:**
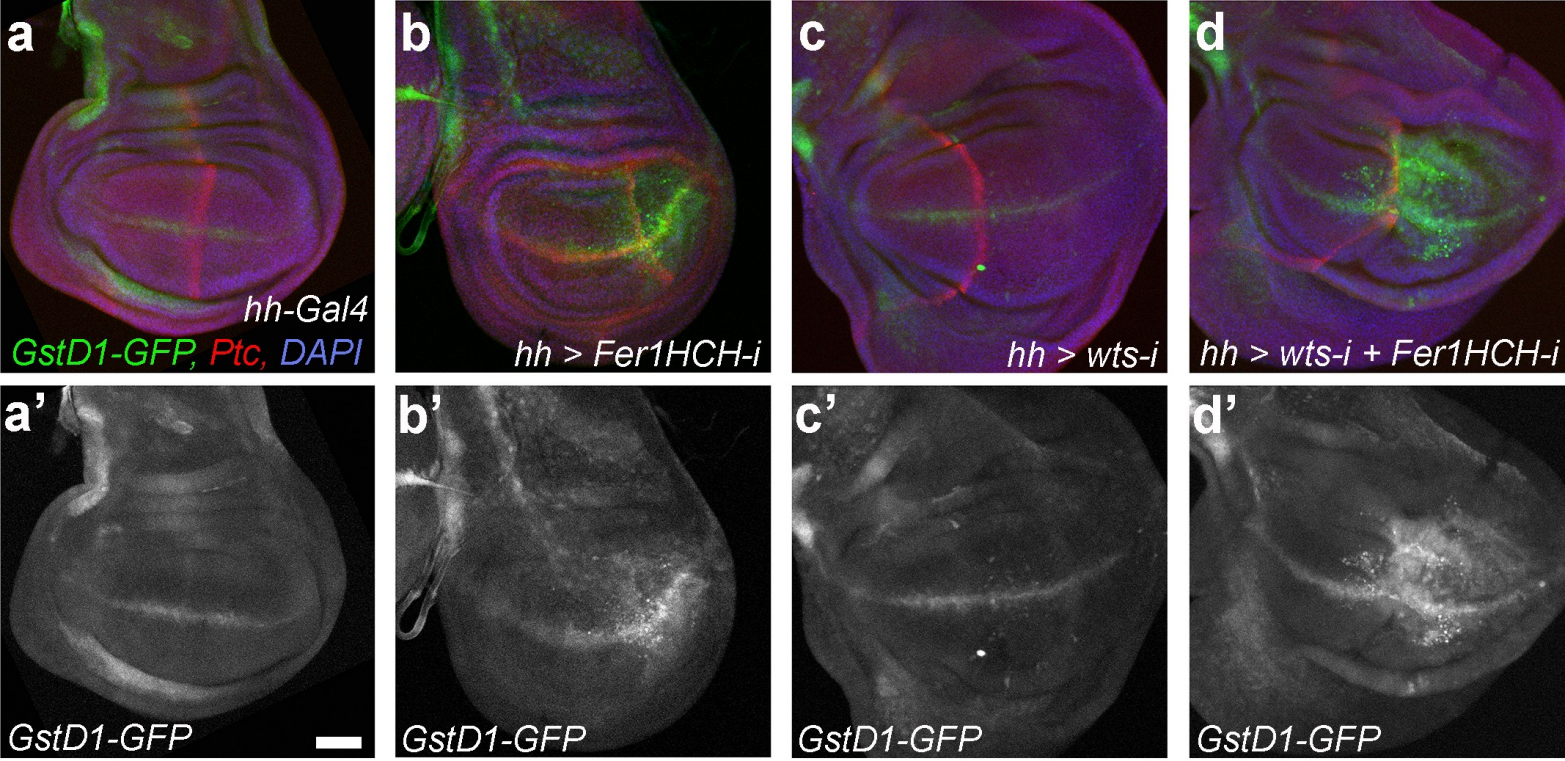
Excess Ferritin in *warts* discs protects against ROS formation. **(a-d’)** Representative wing discs of indicated genotypes. The weaker *Fer1HCH-RNAi* line (49536) was used in these experiments. Dorsal is up, anterior is to the left. *hh-Gal* is expressed in the posterior half of the discs. Ptc (in red) marks the A/P boundary. *GstD1-GFP* reporter indicates ROS accumulation. DAPI labels nuclei. Scale bar in (a’) is 50 microns.

Finally, we tested whether the higher levels of *Fer1HCH* in *wts* mutant discs could influence ROS formation. Therefore, we combined *wts* knockdown with *Fer1HCH* knockdown and assayed for ROS levels. As expected, GstD1-GFP accumulated in the posterior compartment of discs in which we knocked down *Fer1HCH* (Fig 6A and 6B). These discs also had visibly smaller posterior compartments. Notably, discs that express *wts-RNAi* in their posterior compartments did not accumulate ROS despite the increased metabolic activity of these fast-dividing cells (Fig 6C). We wondered whether the elevated *Fer1HCH* transcript levels, and thus likely increased Fer1HCH levels, in *wts* mutant cells had a protective effect. Indeed, simultaneous knockdown of *wts* and *Fer1HCH* led to very high levels of ROS formation (Fig 6D). Overall size of the discs with double *wts* and *Fer1HCH* knockdown was similar to discs with *wts* knockdown despite a relative reduction in the size of the posterior compartment.

## Discussion

Here, we studied the function of the two ferritin subunits in growth control during *Drosophila* larval disc development, a well-established model system for epithelial morphogenesis and tumorigenesis. We found that lowering or increasing levels of the ferritin heavy chain lead to severe mitochondrial defects, which likely ultimately interfere with proper size control. The round and fragmented mitochondria (Fig 4B), along with ROS accumulation (Fig 5) that we found in discs with *Fer1HCH* knockdown are indicative of cells undergoing ferroptosis. This is an exciting finding as the ease of manipulation of imaginal discs coupled to advanced genetic tools in *Drosophila* could highly facilitate the dissection of molecular mechanisms of ferroptosis in an *in vivo* set-up.

The crystal structure of an insect ferritin, from the tiger moth *Trichoplusia ni*, was resolved by Hamburger and colleagues [3]. The structure revealed equal numbers of the heavy and light chains arranged with tetrahedral symmetry. Vertebrate ferritins, on the other hand, contain varying ratios of the heavy and the light chains, depending on the organ [5]. Mice that lack the H Ferritin gene (*Fth*^*-/-*^) die during embryogenesis whereas the deletion of the L Ferritin (*Ftl*^*-/-*^) only cause reduced viability with the majority of the embryos being born without obvious defects [30, 31]. *Ftl*^*-/-*^ animals display iron homeostasis defects upon aging [31]. These observations led to the conclusion that H ferritin homopolymers can sequester iron in mice [31]. Similarly, we suggest that a functional ferritin complex can be achieved in the absence of the light chain in *Drosophila* imaginal discs, even if this is not the physiological form. We found that, *Fer2LCH* knockdown mildly influenced the adult wing size but did not lead to any visible phenotypes in larval tissues. This was true with different drivers. Accordingly, when we overexpressed Fer2LCH or made mosaic tissues with *Fer2LCH*^*35*^, a null allele, both larval and adult tissues had a wild-type appearance. These findings suggest that either Fer1HCH has a role in growth control independent of the ferritin complex or, more likely, that Fer1HCH is the rate-limiting component and has the ability to form a functional complex on its own. Notably, the ferroxidase active site, where iron binds, is found on the heavy chain [5]. There are other examples of the light and the heavy chains having differing effects. When a midgut-specific driver was used, *transferrin 1* knockdown was able to supress eclosion defects of *Fer1HCH* knocked down flies; the treatment increased the eclosion rate from 11% to 36% [32]. However, the same treatment did not rescue the reduced survival of *Fer2LCH* knocked down flies [32]. Another context where the two subunits may behave differently is during blood cell differentiation. *Fer1HCH* knockdown in the intestine led to blood cell differentiation defects, but *Fer2LCH* knockdown did not [33]. Finally, targeting *Fer2LCH* in the clock neurons alters the circadian rhythm, whereas targeting *Fer1HCH* does not [34]. These observations suggest that the two subunits may not strictly function together.

The presence of *Fer1HCH* mutant cells in the discs leads to growth control defects. Surprisingly, we found that *Fer1HCH* mutant cells cannot compete with *Minute* cells, the archetype loser mutation. *Minutes* denote a group of ribosomal mutations with slower growth rates [21]. When the entire organ or organism is *Minute*, the development is delayed but the correct organ size is reached. In mosaics however, *Minute* cells are killed by the wild-type cells, which then populate the whole tissue. The term cell competition was coined to describe this phenomenon [21, 35, 36]. To the best of our knowledge, there has not been any report of a mutation that does not prosper when its clones are induced in a *Minute* background. Thus, mutations in *Fer1HCH* represent the first example of a mutant leading to what we refer to as a “super-loser”.

In our current model, a misbalance in iron metabolism leads to the accumulation of ROS in the tissue, which eventually leads to cell death *via* ferroptosis. Apoptosis also contributes to the observed growth defects, as increased levels of activated caspases were detectable upon *Fer1HCH* knockdown (S3E and S3F Fig). However, preventing apoptosis did not fully rescue these phenotypes (S5 Fig). On the other hand, ROS accumulated in discs where we knocked down *Fer1HCH*, and preventing ROS formation rescued the size defects (Fig 5). Further, treatments that did not influence the tissue size, such as Fer1HCH overexpression, Fer2LCH overexpression, or knockdown of *Fer2LCH*, did not induce ROS accumulation, as monitored by GstD1-GFP. Lastly, there is no excess ROS in discs with reduced *wts* activity, but if *Fer1HCH* is knocked-down simultaneously, very high ROS levels accumulate in the tissue, suggesting a protective role for the excess Fer1HCH in *wts* mutants. Our findings are consistent with the model that a major role of ferritin is its ability to serve as an antioxidant, preventing ROS accumulation [5, 37–40]. It will be interesting to determine whether elevating *Fer1HCH* levels is a general strategy of cancer cells to avoid ferroptosis. Excitingly, a wide range of tumour cells, from liver to prostate, and to osteosarcoma, were shown to be susceptible to ferroptosis, stimulating discussion on using ferroptosis inducers for chemotherapy [41]. However, sensitivity to ferroptosis inducers varies greatly between individuals. Our findings suggest that differences in ferritin expression levels as well as the levels of Hippo activity may underlie some of this variation. Monitoring these parameters may help optimizing treatment options. Strikingly, while our work was under review, a new study directly linked cancer cell ferroptosis to NF2-YAP signalling [42]. Cancer cell lines with high nuclear YAP were found to be more prone to ferroptosis [42]. However, the ferritin levels were not reported. Further work in *Drosophila* will contribute to dissecting out this exciting connection.

## Material and methods

### Fly stocks

Fly stocks were maintained using standard culture conditions. As control strain, *y*,*w* or *w*^*1118*^ flies were used. Mutant alleles *Fer1HCH*^*451*^ and *Fer2LCH*^*35*^ are P(ry[+t7.2] = PZ) insertions and were obtained from the Bloomington Drosophila Stock Center (BDSC); stock numbers are #11497 and #11483, respectively. To induce mosaic clones *Fer1HCH*^*451*^ and *Fer2LCH*^*35*^ were recombined onto FRT82B containing chromosomes. Mosaic clone induction experiments were performed with *ubx-FLP* and *ey-FLP* drivers. *Df(3R)Fer* allele (a.k.a. *Fer*^*x1*^) was a gift from Bertrand Mollereau and is a 2.2 kb deletion disrupting specifically *Fer1HCH* and *Fer2LCH*. We tested all publicly available UAS-RNAi lines for Fer1HCH and Fer2LCH. We provide a summary of the phenotypes obtained using various drivers (Table 1). Crosses were kept at 26°C and discs were dissected at day 5 unless indicated otherwise. Egg collection was limited to 4–6 hours at 26°C to avoid variability in growth and developmental phenotypes for immunohistochemistry.

**Table 1:** Different UAS-RNAi lines tested and a summary of their phenotypes.

UAS-RNAi against	Stock ID	tub-Gal4 phenotype	nub-Gal4 phenotype	ap-Gal4 phenotype	Modify Nub-Gal4 > UAS-Hippo-RNAi phenotype?
Fer1HCH (strong)	VDRC KK12925 or GD102406 (Overlapping target sequence)	Lethal before 3^rd^ instar	Pupal lethal	Pupal lethal	12925 phenotype is dominant, pupal lethal at 25 and 18C
Fer1HCH (weak)	VDRC GD49536 or GD49537 (Identical target sequence)	Lethal before 3^rd^ instar	Smaller wings	Smaller wings	Yes, mild suppression (5%)
Fer1HCH	BL 60000	Lethal before 3^rd^ instar	Very mild (3% reduction)	Smaller wings	No
Fer2LCH	VDRC GD14491	pupal lethal	Smaller wings (14%)		Yes, mild suppression (5%)
Fer2LCH	VDRC KK106960	pupal lethal	Very mild (5% reduction)		Yes
Fer2LCH (strongest)	BL44067	pupal or earlier lethal	Very mild (4% reduction)		Yes, suppression (10%)
Fer2LCH (weakest)	BL60354	semi-lethal, about half can hatch while some die as pupae			

### RT-qPCR analysis

For RNA preparation, wing discs were collected from 12–35 larvae under sterile conditions and immediately lysed. Genotypes used were: wt, day 5 (y w ubxflp/ y w; FRT82B M(3) ubiGFP/ FRT 82B), and *wts*, day 5 and day 9 (y w ubxflp/ y w; FRT82B M(3) ubiGFP/ FRT 82B *wts*^*149*^). RNA extraction was done using Ambion RNAqueous Micro kit. 100ng of RNA was subjected to reverse transcription using the GoScript Reverse Transcriptase Kit (Promega) and 0.5ug of random Primers following the manufacturer’s instructions. Gene expression was determined from cDNA synthesized using GoTaq qPCR Master Mix kit (Promega) using a Fast Real-Time PCR System machine (Applied Biosystems). Relative gene expression was normalized using the housekeeping gene rpr49. Primers used were:

*Fer1HCH* forward: TAATTGCTAGCCTGCTCCTGT

*Fer1HCH* reverse: ATCTCCATAGGCCTGGGC

*Fer2LCH* forward: GCCAGAACACTGTAATCACCG

*Fer2LCH* reverse: GGCTCAATATGGTCAATGCCA

*rpr49* forward: AGCATACAGGCCCAAGATCG

*rpr49* reverse: TGTTGTCGATACCCTTGGGC

### Immunohistochemistry, image analysis and quantifications

Eye or wing imaginal discs were dissected from late wandering 3^rd^ instar larvae in phosphate buffered saline (PBS) and fixed for 25 minutes in 4% Paraformaldehyde at room temperature. Followed by three fast washing steps, samples were washed six times with PBS + 0.1% Triton X-100 (PBT) and blocked for 1h in 3% normal goat serum in PBST (PBTN). Primary antibodies were diluted in PBTN and samples were incubated overnight at 4°C. Followed by two times fast and afterwards six more times washing steps the next day. Secondary antibodies were used at a concentration of 1:750 in PBTN and incubated for 2h at RT. Afterwards samples were rinsed two times fast and washed six times with PBT. Samples were than mounted on slides in Vectashield medium (Vector labs) containing DAPI, if not indicated otherwise. Samples were imaged using a Zeiss LSM 880 and images were processed using ImageJ, and figures were prepared using Adobe software. Pictures of the adult wings were taken on a Leica DMI6000 stereomicroscope. Statistical analysis and quantifications were done with R studio and Excel.

### EdU staining

Click-IT EdU Alexa-Fluor 594 staining kit from Invitrogen was used to detect cell proliferation. Larvae were washed prior to dissection three times in cold PBS. Dissection was performed in cold Schneider’s Drosophila Medium (Gibco). Afterwards samples were incubated in 15uM EdU diluted in Schneider’s Medium + 1% Normal Goat Serum for 15min at RT in the dark on a shaker. Then, samples were washed two times with 3% BSA, followed by the fixation step with 4% PFA (in PBS) for 20min at RT on a shaker. After removal of the fixative, samples were washed twice with 3% BSA and then 3 times fast, followed by a 20min washing step with 0.5% Triton X-100 in PBS. Before adding the Click-IT reaction cocktail for 30min, samples were washed two times with 3% BSA again. Finally, the reaction cocktail was removed and samples were washed three times with 3% BSA followed by 30min washing steps with PBS, before adding the Vectashield mounting medium.

### TUNEL staining

To detect apoptotic cells the in situ cell death detection kit, TMR red from Roche was used. Terminal deoxynucleotidyl transferase dUTP Nick-End labelling (TUNEL) staining was performed according to manufacturer’s protocol on dissected larvae.

### TEM sample preparation

Wing discs were dissected in PBS and immediately fixed with 1% paraformaldehyde, 2.5% glutaraldehyde in 0.1M cacodylate buffer for two hours at room temperature. The samples were incubated for 1 hour in 2% (wt/vol) osmium tetroxide and 1.5% (wt/vol) K_4_[Fe(CN)_6_] in cacodylate buffer followed by 1 hour in 1% (wt/vol) tannic acid in 100 mM cacodylate buffer, then 30 minutes in 2% (wt/vol) Osmium tetroxide followed by 1% (wt/vol) uranyl acetate for 2h at room temp. After the dehydration cycles, and incubations in Epon-Araldite mix, samples were flat embedded and cured for 24h at 60°C [43, 44].

### TEM observation and analysis

Polymerized flat blocks were trimmed using 90° diamond trim tool (Diatome, Switzerland) and using 35° diamond knife (Diatome, Switzerland) mounted on Leica UC6 microtome (Leica, Austria), 70 nm sections were collected on formvar-coated slot grids (EMS, USA). For sectioning, samples were carefully oriented to obtain the relevant parts of the wing blade, indispensable for the reliable data interpretation [43, 45]. TEM samples were analyzed with an FEI CM100 electron microscope operated at 80kV, equipped with TVIPS camera, piloted by EMTVIPS program. Images were collected either as single frames or stitched mosaic panels to cover larger regions of the sample. Data were processed and analyzed using Fiji, IMOD 3dmod and Photoshop programs.

## Supporting information

S1 Fig The expression of the Ferritin complex is induced in *warts* mutants. **(a)** Row normalized heat-maps of top 15 and bottom 5 of the 120 genes that are induced in *warts (wts)* mutant discs with a cut-off of 2,3-fold induction. The color key is coded in log2. Green bars represent average expression levels. **(b-c)** RT-qPCR analysis of (b) *Fer1HCH* and (c) *Fer2LCH* mRNA levels in wing discs that are nearly fully mutant for a null *wts* allele at day 5 and day 9. In such discs, *wts* mutant cells take over the disc and cause tissue overgrowth. The genotypes are:
*control*: *y ubx-flp / y w; FRT82B M(3) ubiGFP / FRT82B* at day 5
*wts*: *y ubx-flp / y w; FRT82B M(3) ubiGFP / FRT82B wts*^*149*^ at day 5 and day 9.
(TIF)

S2 Fig Targeting the Ferritin complex modifies wing growth. **(a)** *Nub-Gal4* driven knockdown of *Fer1HCH* and *Fer2LCH* using all available UAS-RNAi lines from the Vienna Drosophila Resource Center (VDRC). Note that the two strong RNAi lines (102406 and 12925) cause lethality at pupal stage and the few escapers have no wings. Representative adult wings and quantifications of wing areas for the indicated genotypes are shown. **(b)** Knockdown of *Fer1HCH* and *Fer2LCH* modify the overgrowth phenotype of *Nub-Gal4 > UAS-hippo-RNAi* wings to differing extents. Statistical significance is indicated as ns: p>0.05, *: p≤0.05, **: p≤0.01, ***: p≤0.001, ****: p≤ 0.0001.
(TIF)

S3 Fig Cell proliferation and apoptosis profiles upon knockdown of the heavy and the light chains. **(a-d’)** EdU (purple in a-d and gray in a’-d’) profiles upon knockdown of *Fer1HCH* with (b) a weak (VDRC 49537) and (c) a stronger (VDRC 102406) RNAi line in the posterior compartments (right hand side). (d) *Fer2LCH* knockdown in the posterior compartment does not influence the EdU pattern. Ptc antibody staining in green marks the A/P boundary. **(e-h’)** Activated caspase expression revealed by anti-Dcp-1 (purple in e-h and gray in e’-h’) in discs with *hh-gal4* (posterior specific) driven knockdown of *Fer1HCH* with (f) a weak (VDRC 49537) and (g) a stronger (VDRC 102406) RNAi line. (h) *Fer2LCH* knockdown in the posterior compartment does not induce caspase activation. Ptc antibody staining in green marks the A/P boundary. **(i)** *TUNEL* staining in day 5 discs mosaic for *Fer1HCH*^*451*^ (two discs are shown), *Fer2LCH*^*35*^, and the *Df(3R)Fer* deletion. GFP (green) marks the Minute cells and *Fer* mutant cells are unmarked. *TUNEL* is shown separately in gray below each panel. Apoptosis is induced in discs mosaic for the heavy chain in a non-cell-autonomous and variable fashion. The genotypes are:
*wt*: *y ubx-flp / y w; FRT82B M(3) ubiGFP / FRT82B*
*fer1*: *y ubx-flp / y w; FRT82B M(3) ubiGFP / FRT82B Fer1HCH*^*451*^
*fer2*: *y ubx-flp / y w; FRT82B M(3) ubiGFP / FRT82B Fer2LCH*^*35*^
*Df(3R)*: *y ubx-flp / y w; FRT82B M(3) ubiGFP / FRT82B Df(3R)Fer*.
(TIF)

S4 Fig Clonal phenotypes described for the wing disc are not tissue specific. Representative eye discs of indicated genotypes at day 5. All discs are shown at the same scale. Scale bar in (h) is 50 microns. (i and j) show quantifications of area occupied by the mutant cells as a percentage of the whole disc area. Genotypes are:
a) *y eyflp; FRT82B ubiGFP / FRT82B*
b) *y eyflp; FRT82B ubiGFP / FRT82B Fer1HCH*^*451*^
c) *y eyflp; FRT82B ubiGFP / FRT82B Fer2LCH*^*35*^
d) *y eyflp; FRT82B ubiGFP / FRT82B Df(3R)Fer*
e) *y eyflp; FRT82B M(3) ubiGFP / FRT82B*
f) *y eyflp; FRT82B M(3) ubiGFP / FRT82B Fer1HCH*^*451*^
g) *y eyflp; FRT82B M(3) ubiGFP / FRT82B Fer2LCH*^*35*^
h) *y eyflp; FRT82B M(3) ubiGFP / FRT82B Df(3R)Fer*.
(TIF)

S5 Fig Cell clones with *Fer1HCH* knockdown are eliminated from the tissue. **(a-e)** Representative third instar wing discs with flp-out clones expressing (a) *UAS-GFP*, (b) *UAS-GFP + UAS-Fer1HCH-RNAi-s (VDRC102406)*, (c) *UAS-GFP + UAS-Fer1HCH-RNAi-s + UAS-DIAP1*, (d) *UAS-GFP + UAS-Fer1HCH- RNAi-s + UAS-p35*, and (e) *UAS-GFP + UAS-Fer1HCH- RNAi-s + UAS-Yki*. GFP (green) marks the modified clonal patches and DAPI (blue) labels nuclei. All samples were treated in parallel and imaged at the same settings. **(f)** Shows quantifications of (left) clone occupancy (% GFP-positive area / total disc area), and (right) overall disc area normalized to the average control disc size. Five discs per genotype were measured; error bars represent standard deviation. The rescue by UAS-Yki (e vs b) is significant (p≤0.001). The rescue by UAS-DIAP1 and p35 (c,d vs b) are merely significant (p≤0.05).
(TIF)

S6 Fig Architecture of *ptc > Fer1HCH-RNAi* discs are heavily disturbed. **(a-c)** Apical to basal cross-section of tiled TEM images of representative discs with *ptc-Gal4* driven expression of indicated UAS-RNAi lines. (a) Discs expressing UAS-Fer2LCH have an overall wild-type appearance. (b) Discs with *Fer1HCH* (VDRC 102406) knockdown are thinner and disorganized. (c) Discs with ectopic Fer1HCH look nearly normal. All TEM composites (a-c) are shown at the same magnification, scale bar in (b) is 2nm. **(d)** Representative third instar discs of indicated genotypes used for TEM analysis, shown at the same magnification. Scale bar is 50uM. **(e-f)** Shows examples of mitochondria that were classified as normal (e) or abnormal (f) for quantification shown in Fig 4G.
(TIF)
