# Correction: Ferritin heavy chain protects the developing wing from reactive oxygen species and ferroptosis

**DOI:** 10.1371/journal.pgen.1009138

**Published:** 2020-10-15

**Authors:** Simone Mumbauer, Justine Pascual, Irina Kolotuev, Fisun Hamaratoglu

The labelling on the x-axis in Fig 5J is incorrect. The authors have provided a corrected version here.

**Fig 5:**
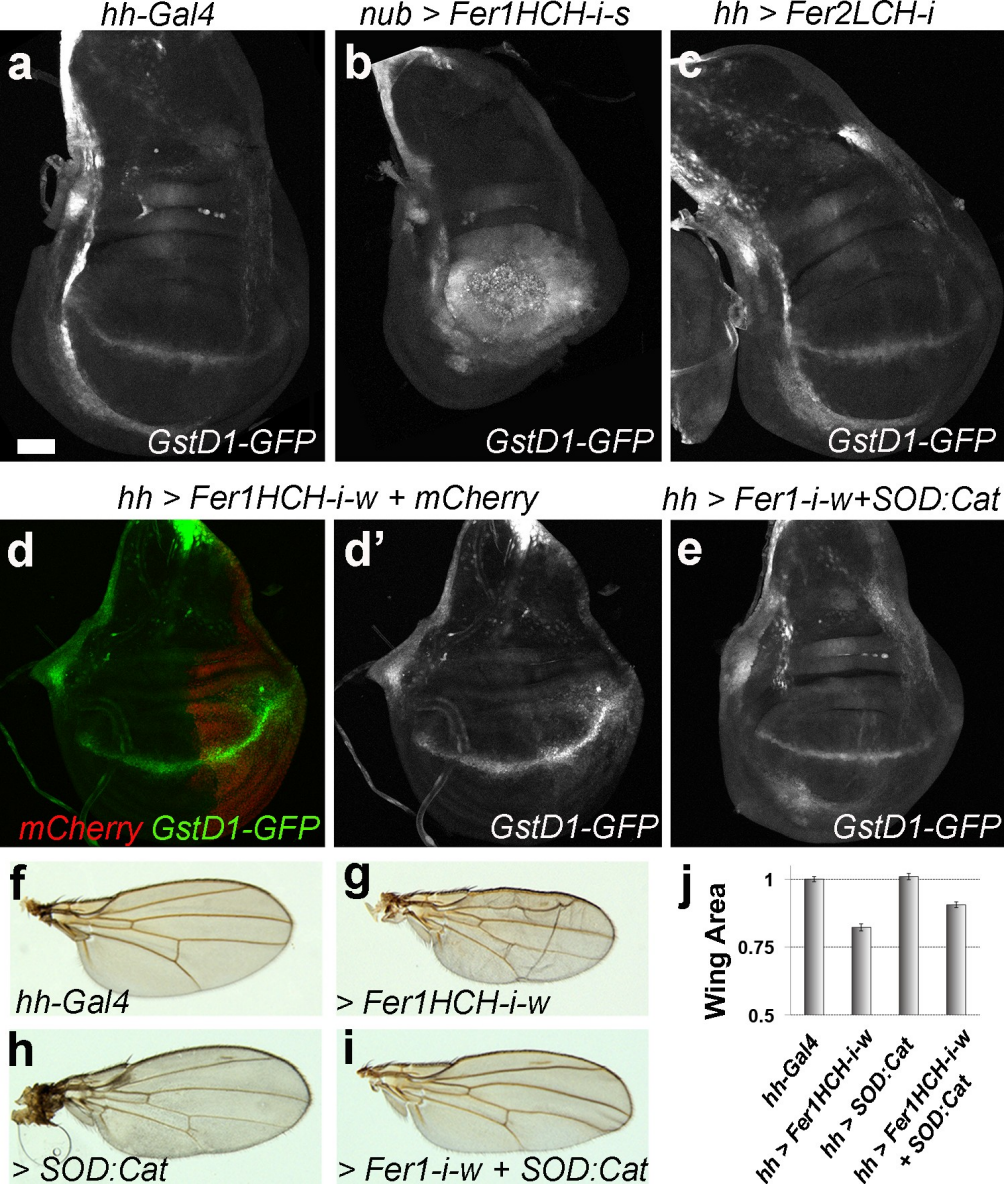
Low levels of the Ferritin heavy chain cause ROS accumulation. (a-e) Representative wing discs of indicated genotypes. Dorsal is up, anterior is to the left. hh-Gal is expressed in the posterior compartment and nub-Gal4 is expressed in the pouch (used in panel b). (d) shows the GstD1-GFP signal alone of the disc shown in panel (d). All discs are from day 5 larvae with the exception of panel (b), which shows a day 8 disc. (f-i) Representative wings of indicated genotypes and (j) quantification of their areas normalized to control wings (minimum 10 wings per genotype) are shown. Error bars represent standard error. P-values for t-tests are: hh-Gal4 vs Fer1HCH-i-w (p≤ 0.0001); hh-Gal4 vs Fer1HCH-i-w + SOD:Cat (p≤ 0.01); Fer1HCH-i-w vs Fer1HCH-i-w + SOD:Cat (p≤ 0.001). All discs and all wings are shown at the same scale. Scale bars in (a) and (f) are 50 and 100 microns respectively.
