# Supplementary figures and images for: Ferritin heavy chain protects the developing wing from reactive oxygen species and ferroptosis
Source: PLoS Genet. 2019 Sep 30;15(9):e1008396. doi: 10.1371/journal.pgen.1008396 (PMC6786644; doi:10.1371/journal.pgen.1008396)

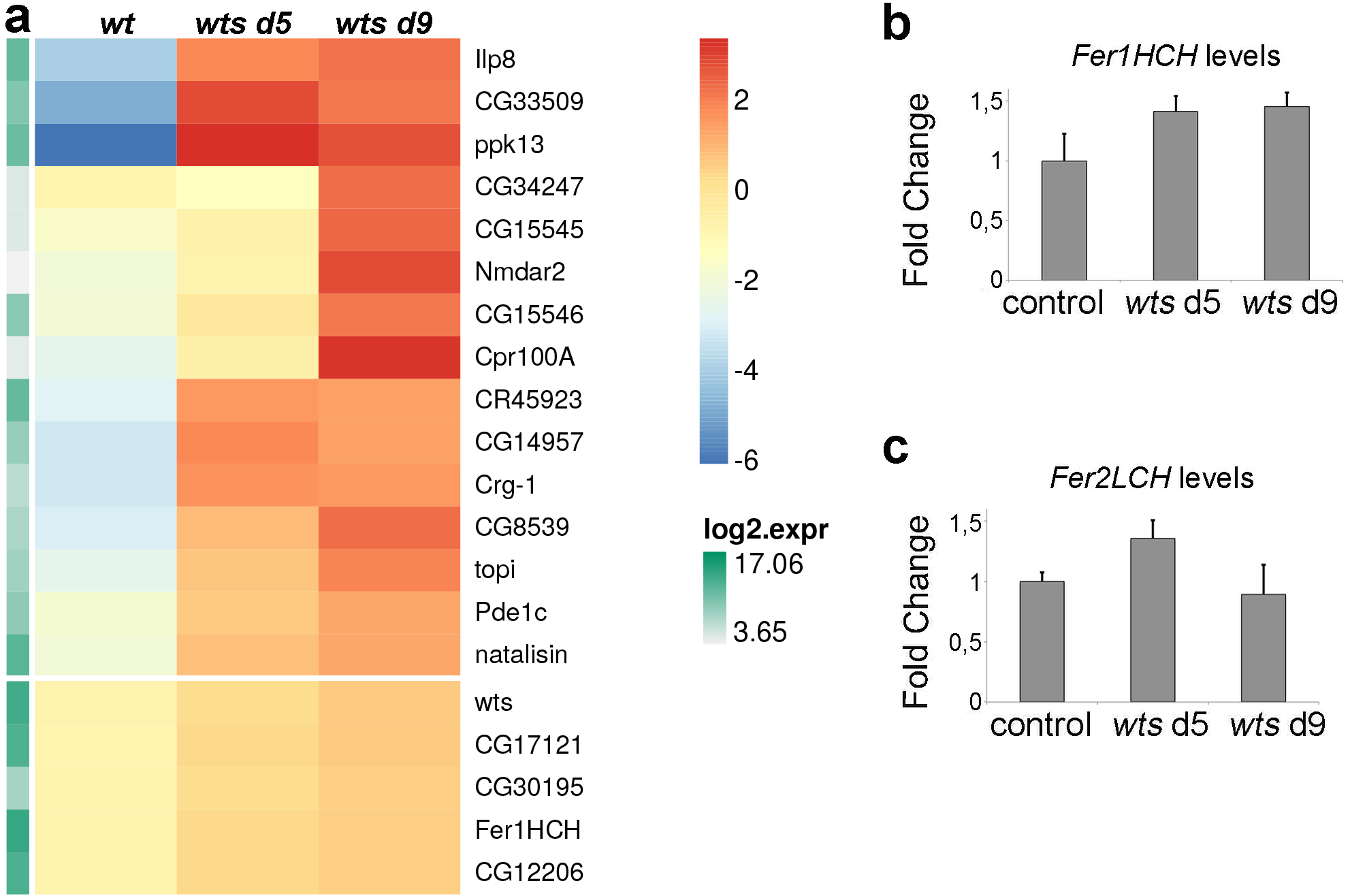

Supplement: S1 Fig — (a) Row normalized heat-maps of top 15 and bottom 5 of the 120 genes that are induced in warts (wts) mutant discs with a cut-off of 2,3-fold induction. The color key is coded in log2. Green bars represent average expression levels. (b-c) RT-qPCR analysis of (b) Fer1HCH and (c) Fer2LCH mRNA levels in wing discs that are nearly fully mutant for a null wts allele at day 5 and day 9. In such discs, wts mutant cells take over the disc and cause tissue overgrowth. The genotypes are: control: y ubx-flp / y w; FRT82B M(3) ubiGFP / FRT82B at day 5 wts: y ubx-flp / y w; FRT82B M(3) ubiGFP / FRT82B wts149 at day 5 and day 9. (TIF) [file pgen.1008396.s001.tif]

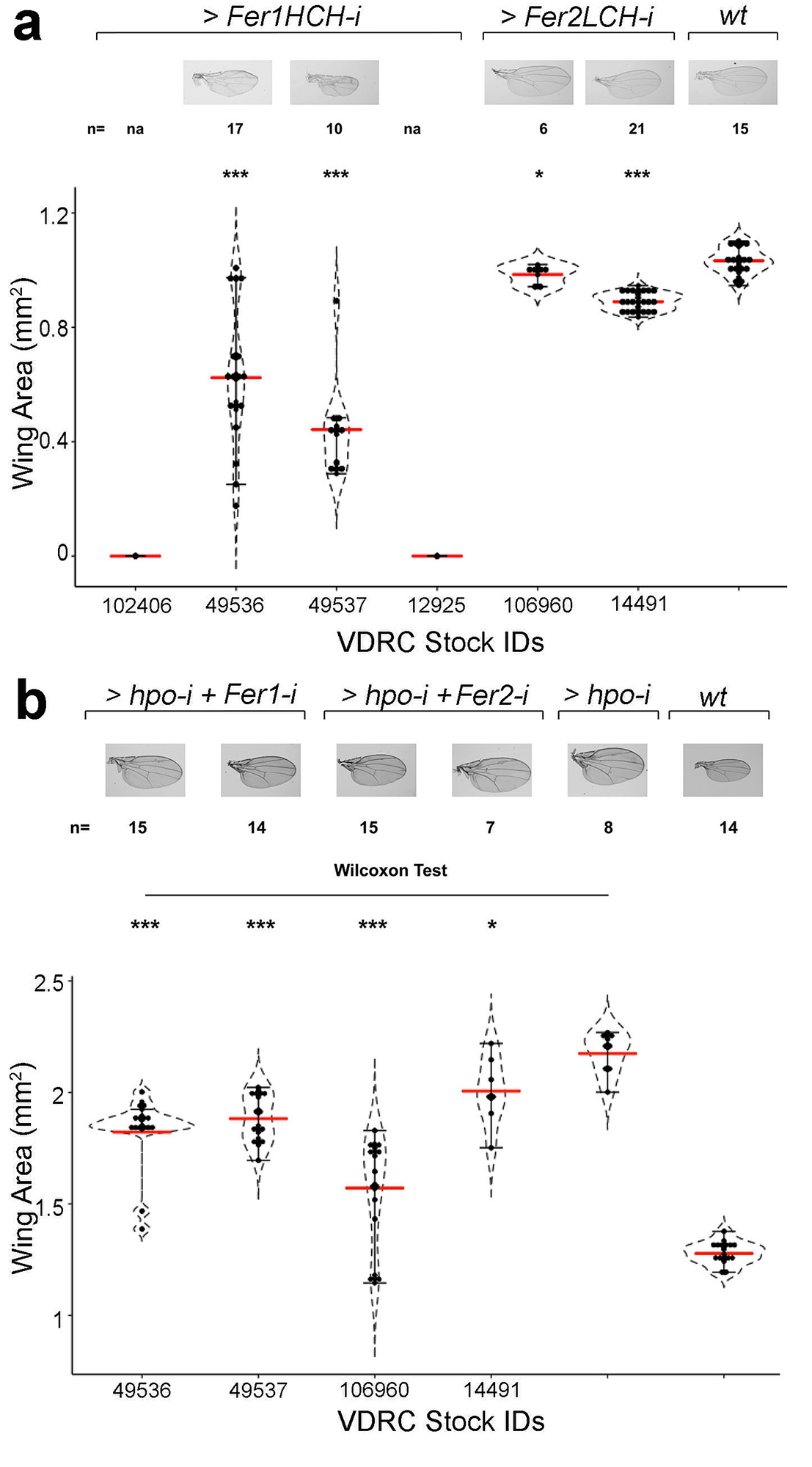

Supplement: S2 Fig — (a) Nub-Gal4 driven knockdown of Fer1HCH and Fer2LCH using all available UAS-RNAi lines from the Vienna Drosophila Resource Center (VDRC). Note that the two strong RNAi lines (102406 and 12925) cause lethality at pupal stage and the few escapers have no wings. Representative adult wings and quantifications of wing areas for the indicated genotypes are shown. (b) Knockdown of Fer1HCH and Fer2LCH modify the overgrowth phenotype of Nub-Gal4 > UAS-hippo-RNAi wings to differing extents. Statistical significance is indicated as ns: p>0.05, *: p≤0.05, **: p≤0.01, ***: p≤0.001, ****: p≤ 0.0001. (TIF) [file pgen.1008396.s002.tif]

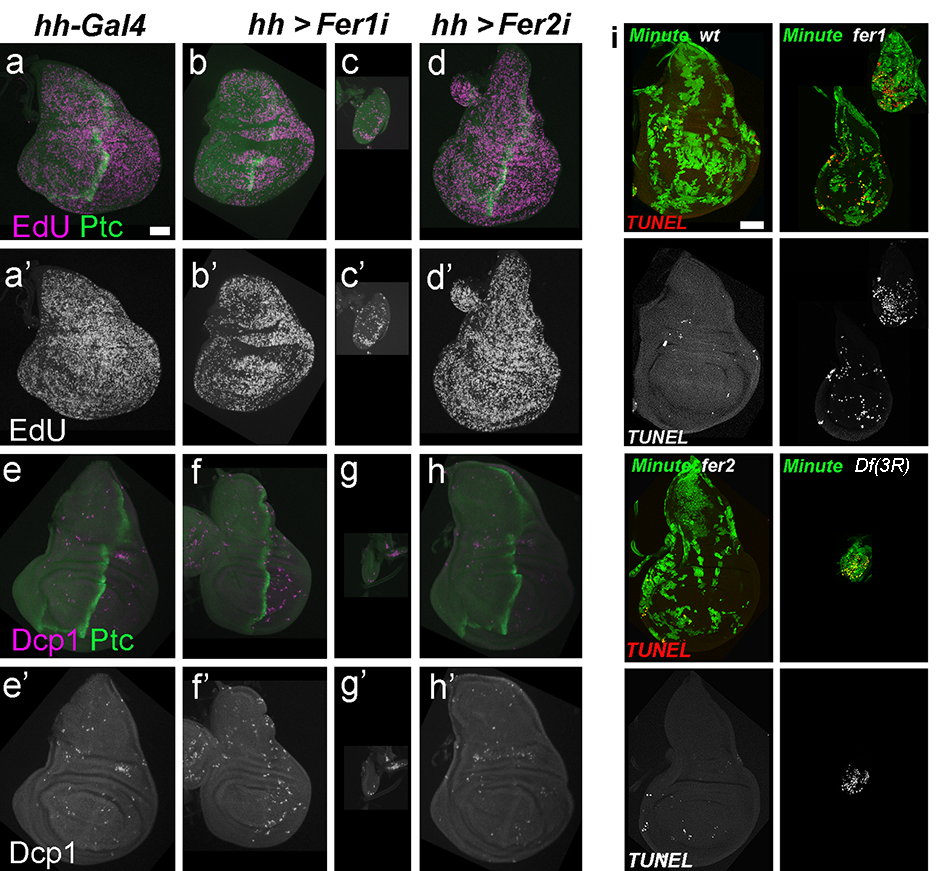

Supplement: S3 Fig — (a-d’) EdU (purple in a-d and gray in a’-d’) profiles upon knockdown of Fer1HCH with (b) a weak (VDRC 49537) and (c) a stronger (VDRC 102406) RNAi line in the posterior compartments (right hand side). (d) Fer2LCH knockdown in the posterior compartment does not influence the EdU pattern. Ptc antibody staining in green marks the A/P boundary. (e-h’) Activated caspase expression revealed by anti-Dcp-1 (purple in e-h and gray in e’-h’) in discs with hh-gal4 (posterior specific) driven knockdown of Fer1HCH with (f) a weak (VDRC 49537) and (g) a stronger (VDRC 102406) RNAi line. (h) Fer2LCH knockdown in the posterior compartment does not induce caspase activation. Ptc antibody staining in green marks the A/P boundary. (i) TUNEL staining in day 5 discs mosaic for Fer1HCH451 (two discs are shown), Fer2LCH35, and the Df(3R)Fer deletion. GFP (green) marks the Minute cells and Fer mutant cells are unmarked. TUNEL is shown separately in gray below each panel. Apoptosis is induced in discs mosaic for the heavy chain in a non-cell-autonomous and variable fashion. The genotypes are: wt: y ubx-flp / y w; FRT82B M(3) ubiGFP / FRT82B fer1: y ubx-flp / y w; FRT82B M(3) ubiGFP / FRT82B Fer1HCH451 fer2: y ubx-flp / y w; FRT82B M(3) ubiGFP / FRT82B Fer2LCH35 Df(3R): y ubx-flp / y w; FRT82B M(3) ubiGFP / FRT82B Df(3R)Fer. (TIF) [file pgen.1008396.s003.tif]

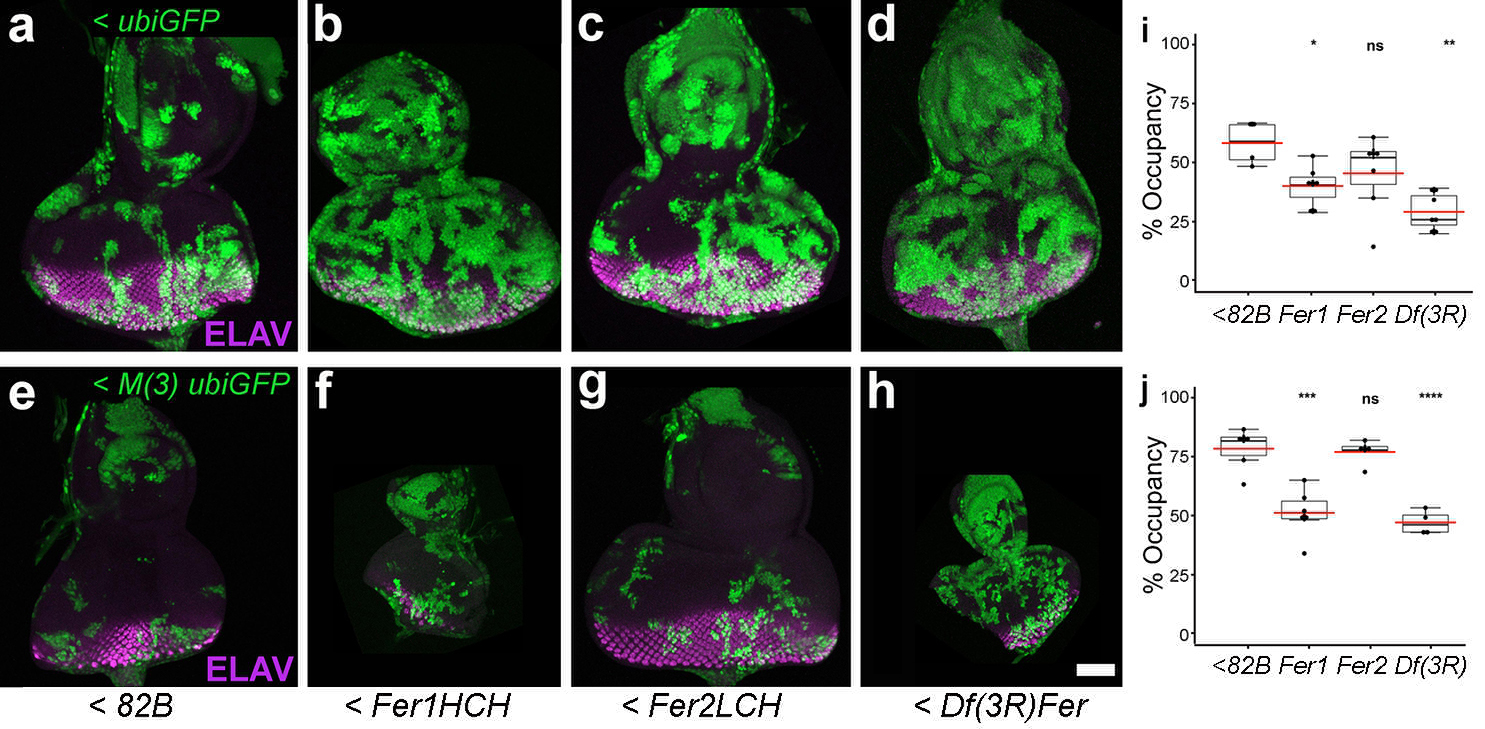

Supplement: S4 Fig — Representative eye discs of indicated genotypes at day 5. All discs are shown at the same scale. Scale bar in (h) is 50 microns. (i and j) show quantifications of area occupied by the mutant cells as a percentage of the whole disc area. Genotypes are: a) y eyflp; FRT82B ubiGFP / FRT82B b) y eyflp; FRT82B ubiGFP / FRT82B Fer1HCH451 c) y eyflp; FRT82B ubiGFP / FRT82B Fer2LCH35 d) y eyflp; FRT82B ubiGFP / FRT82B Df(3R)Fer e) y eyflp; FRT82B M(3) ubiGFP / FRT82B f) y eyflp; FRT82B M(3) ubiGFP / FRT82B Fer1HCH451 g) y eyflp; FRT82B M(3) ubiGFP / FRT82B Fer2LCH35 h) y eyflp; FRT82B M(3) ubiGFP / FRT82B Df(3R)Fer. (TIF) [file pgen.1008396.s004.tif]

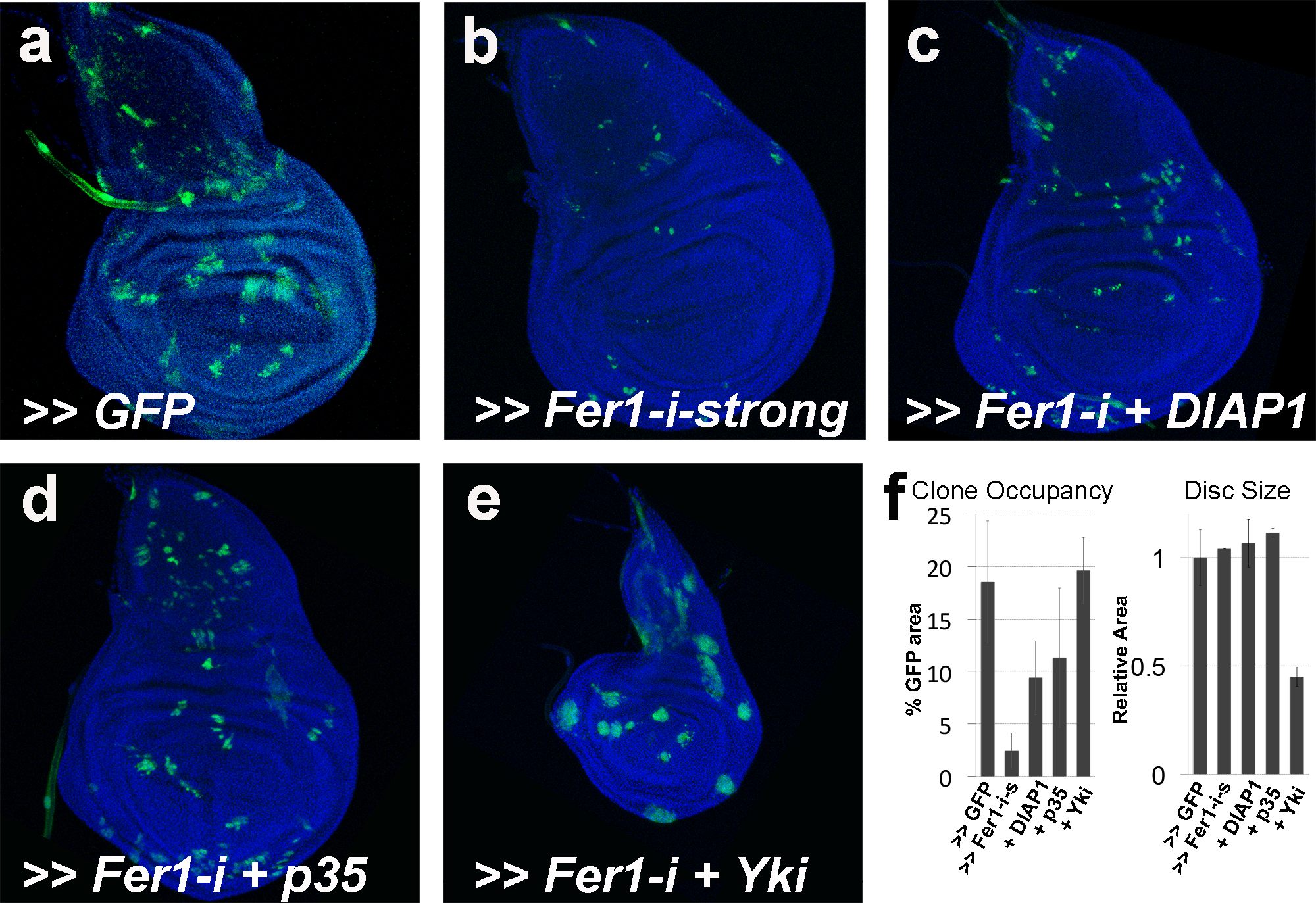

Supplement: S5 Fig — (a-e) Representative third instar wing discs with flp-out clones expressing (a) UAS-GFP, (b) UAS-GFP + UAS-Fer1HCH-RNAi-s (VDRC102406), (c) UAS-GFP + UAS-Fer1HCH-RNAi-s + UAS-DIAP1, (d) UAS-GFP + UAS-Fer1HCH- RNAi-s + UAS-p35, and (e) UAS-GFP + UAS-Fer1HCH- RNAi-s + UAS-Yki. GFP (green) marks the modified clonal patches and DAPI (blue) labels nuclei. All samples were treated in parallel and imaged at the same settings. (f) Shows quantifications of (left) clone occupancy (% GFP-positive area / total disc area), and (right) overall disc area normalized to the average control disc size. Five discs per genotype were measured; error bars represent standard deviation. The rescue by UAS-Yki (e vs b) is significant (p≤0.001). The rescue by UAS-DIAP1 and p35 (c,d vs b) are merely significant (p≤0.05). (TIF) [file pgen.1008396.s005.tif]

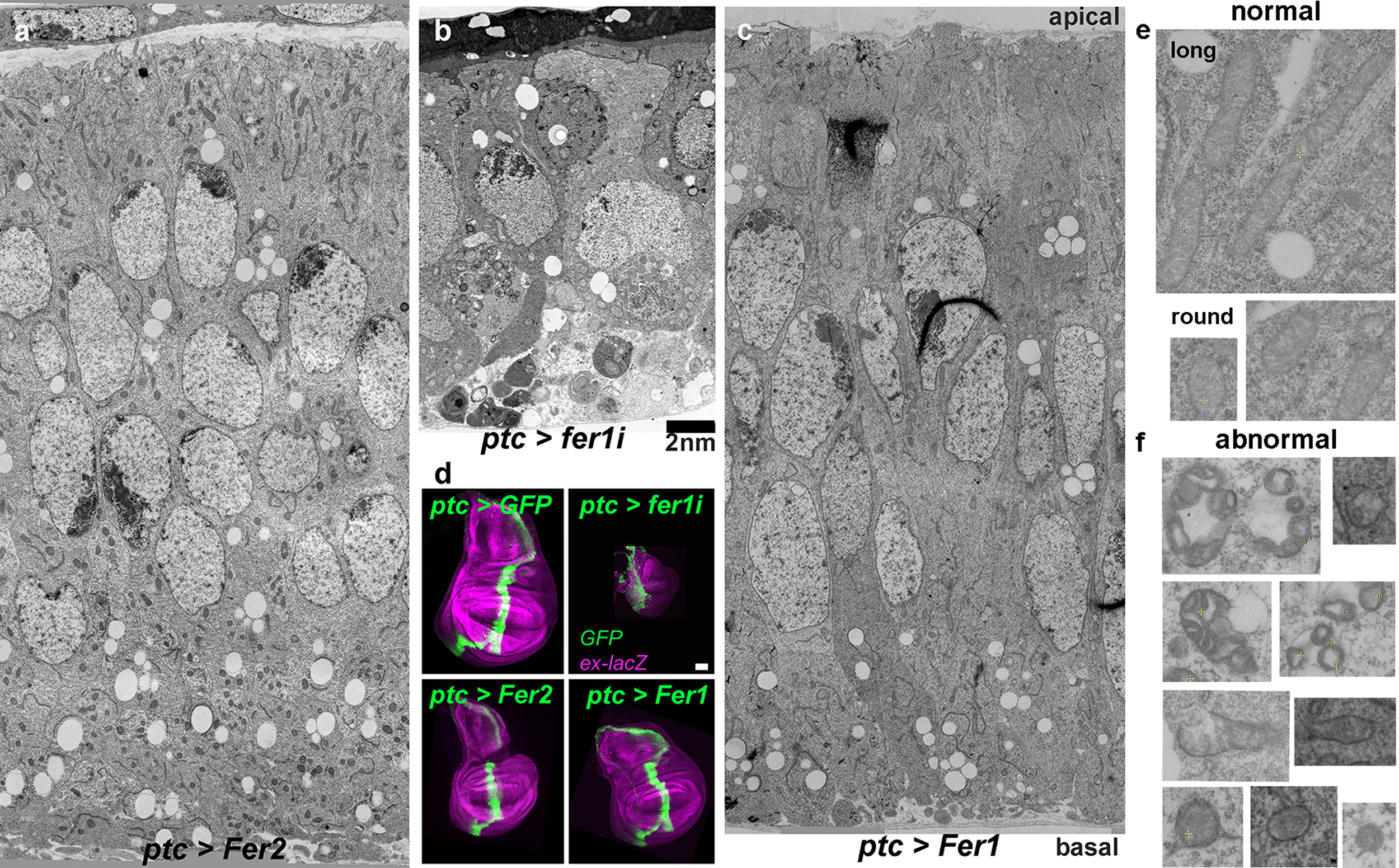

Supplement: S6 Fig — (a-c) Apical to basal cross-section of tiled TEM images of representative discs with ptc-Gal4 driven expression of indicated UAS-RNAi lines. (a) Discs expressing UAS-Fer2LCH have an overall wild-type appearance. (b) Discs with Fer1HCH (VDRC 102406) knockdown are thinner and disorganized. (c) Discs with ectopic Fer1HCH look nearly normal. All TEM composites (a-c) are shown at the same magnification, scale bar in (b) is 2nm. (d) Representative third instar discs of indicated genotypes used for TEM analysis, shown at the same magnification. Scale bar is 50uM. (e-f) Shows examples of mitochondria that were classified as normal (e) or abnormal (f) for quantification shown in Fig 4G. (TIF) [file pgen.1008396.s006.tif]
